# NINJ1-mediated plasma membrane rupture of pyroptotic endothelial cells exacerbates blood-brain barrier destruction caused by neutrophil extracellular traps in traumatic brain injury

**DOI:** 10.1038/s41420-025-02350-x

**Published:** 2025-02-20

**Authors:** Xiao-Bo Zheng, Xue Wang, Sheng-Qing Gao, Chao-Chao Gao, Tao Li, Yan-Ling Han, Ran Zhao, Yan Sun, Shu-Hao Miao, Jia-Yin Qiu, Wang-Xuan Jin, Meng-Liang Zhou

**Affiliations:** 1 Jinling Clinical Medical College, Nanjing University of Chinese Medicine, Nanjing, China; 2 Department of Neurosurgery, Nanjing Jinling Hospital, Affiliated Hospital of Medical School, Nanjing University, Nanjing, China; 3 Department of Neurosurgery, Jinling Hospital, Jinling School of Clinical Medicine, Nanjing Medical University, Nanjing, China

**Keywords:** Neutrophils, Blood-brain barrier, Brain injuries, Cell death, Cell death in the nervous system

## Abstract

Brain endothelial cell (bEC) dysfunction is the main factor of blood-brain barrier (BBB) breakdown, which triggers a vicious cycle of aggravating traumatic brain injury (TBI) pathogenesis. Previous studies have revealed that neutrophil extracellular traps (NETs) released by neutrophils can lead to BBB disruption, but there is a lack of research on the underlying mechanisms after TBI. Here, excessive NETs were found in both contused brain tissue and circulation following TBI. We found that NETs could activate the TLR4/NF-κB pathway to induce bEC pyroptosis, which led to BBB disruption after TBI. During this process, ninjurin-1 (NINJ1) was activated in pyroptotic bECs, and it mediated the release of high mobility group box 1 protein (HMGB1) via plasma membrane rupture (PMR) to promote NET formation. NINJ1-mediated release of HMGB1 aggravated NET accumulation by forming a vicious circle following TBI. Knockdown of NINJ1 rescued NET formation, attenuated BBB leakage, and improved neurological outcomes after TBI. NINJ1 may represent a promising target for alleviating NET-induced BBB destruction and other related injuries after TBI.

## Introduction

In recent years, traumatic brain injury (TBI), caused by an external mechanical force to the brain, usually a violent blow, has emerged as a major public health concern due to its high mortality and morbidity rates [1, 2]. Following the impact of the external force, the acceleration-deceleration movement of brain tissue leads to the shearing and tearing of cerebrovascular systems, neurons, and glia, which is known as primary injury [3]. Cellular dysfunction triggered by mechanical insult leads to secondary cellular injuries, such as mitochondrial dysfunction, neuroinflammation, excitotoxicity, calcium overload, and oxidative stress, which contribute to blood-brain barrier (BBB) damage, ischemia, hypoxia, and increased risk of multiple neurological disease, such as stoke [3–6]. Elaborate tight junctions composed of occludin, claudin, and zonula occludens (ZO) family members are located between adjacent brain endothelial cells (bECs) and form a selective barrier between the brain and the periphery that highly regulates the central nervous system (CNS) internal milieu [7, 8]. The leaky BBB exposes the brain to harmful blood components, which aggravates neuroinflammation and increases neuronal cell death, causes the formation of cytotoxic and vasogenic brain edema, and eventually raises intracranial pressure, which is closely associated with poor neurological outcomes following TBI [9]. Although many research efforts have focused on BBB dysfunction after TBI, more research is needed to provide more therapeutic options for early intervention.

Neutrophils are the most abundant circulating leukocytes in mammals and are the frontline immune cells that fight infection and injury [10]. During TBI onset, a large number of neutrophils flood into the lesion, and excessive neutrophil infiltration has been reported in multiple pathological mechanisms after TBI, which can intensify the inflammatory response and collapse of the neurovascular unit, ultimately resulting in BBB dysfunction [11, 12]. In 2004, Volker Brinkmann et al. first reported that neutrophils can release a type of extracellular web-like structure formed by myeloperoxidase (MPO), neutrophil elastase (NE), histones, and other substances assembled on double-stranded DNA (dsDNA) called neutrophil extracellular traps (NETs) [13]. Peptidylarginine deiminase 4 (PAD4) plays an important role during NET formation, which leads to chromatin dedensification via catalyzing the citrullination of histones and promotes the extrusion of NETs [14]. In addition to degranulation and phagocytosis, NETs are considered an important defensive mechanism for neutrophils to eliminate pathogens [15]. However, excessive NETs can aggravate inflammation, promote thrombosis, and lead to vascular injury, which is involved in the regulation of various diseases, such as autoimmune disorders, ischemia-reperfusion injury, and the development of fibrosis [16]. Similarly, NETs mediate various pathogenesis following TBI, including endoplasmic reticulum stress, coagulopathy, neuroinflammation, neuronal apoptosis, and cerebral edema, all of which contribute to neurological deterioration [17–19]. Importantly, BBB breakdown is a major cause of NET-induced neurological deficits after TBI. However, the molecular mechanisms responsible for NET-induced BBB destruction following TBI are poorly understood.

Pyroptosis, a novel form of inflammatory programmed cell death, is characterized by gasdermin D (GSDMD)-driven cell expansion, cytomembrane pore formation, and the release of intracellular proinflammatory cytokines such as IL-1β and IL-18 [20]. GSDMD is a pore-forming protein and the ultimate executor of pyroptosis [21]. A recent study showed that GSDMD is highly expressed in bECs. When bECs undergo pyroptosis, the pores perforated activated GSDMD are not only used for the release of inflammatory factors but also increase the permeability of the cerebrovascular barrier [20, 22]. Moreover, bECs are a vital component of the BBB, and the pyroptosis of bECs directly disrupts the integrity of the BBB [23]. Therefore, attenuating the GSDMD-dependent pyroptosis of bECs in pathological conditions could be an effective intervention to alleviate BBB leakage. It has been reported that multiple components of NETs can be recognized as the ligands for toll-like receptor 4 (TLR4). Studies on sepsis have shown that NETs lead to endothelial cell dysfunction via activation of the TLR4/nuclear factor kappa-B (NF-κB) pathway, promoting the transcription of inflammatory factors and the transformation of endothelial cells into an inflammatory phenotype [24]. However, there is no corresponding study on the relationship between the NET-activated TLR4/NF-κB pathway and bEC pyroptosis.

Nerve injury-induced protein 1 (Ninjurin-1, NINJ1) was initially discovered as an adhesion molecule upregulated in Schwann cells after peripheral nerve injury [25]. A recent study revealed that NINJ1 is the final effector molecule mediating plasma membrane rupture (PMR) and the diffusion of inflammatory factors during lytic cell death [26]. Intracellular contents known as damage-associated molecular patterns (DAMPs) are a series of molecules released from necrotic cells that can spread danger signaling during tissue damage to recruit immune cells [27]. As a representative DAMP, high mobility group box 1 protein (HMGB1) is widely involved in the regulation of the inflammatory response. Because HMGB1 is a large DAMP, it cannot pass through GSDMD pores, but it must be released via PMR [28, 29]. HMGB1 has also been reported to be a potent activator of NET formation [30]. Therefore, we wondered whether NINJ1 regulates the PMR of pyropotic bECs, thereby promoting NET generation, which further aggravates damage after TBI.

In this study, we investigated the mechanism underlying NET-induced BBB breakdown after TBI. We assumed that NETs could increase the permeability of the cerebrovascular barrier by promoting bEC pyroptosis after TBI, and this effect was mediated by the TLR4/NF-κB pathway. Then NINJ1-mediated PMR of pyroptotic bECs in turn promoted neutrophil infiltration and NET formation, which led to further bEC pyroptosis, BBB disruption, and poor neurological outcomes after TBI.

## Results

### NETs are induced around cerebral vessels after TBI

**Fig. 1:**
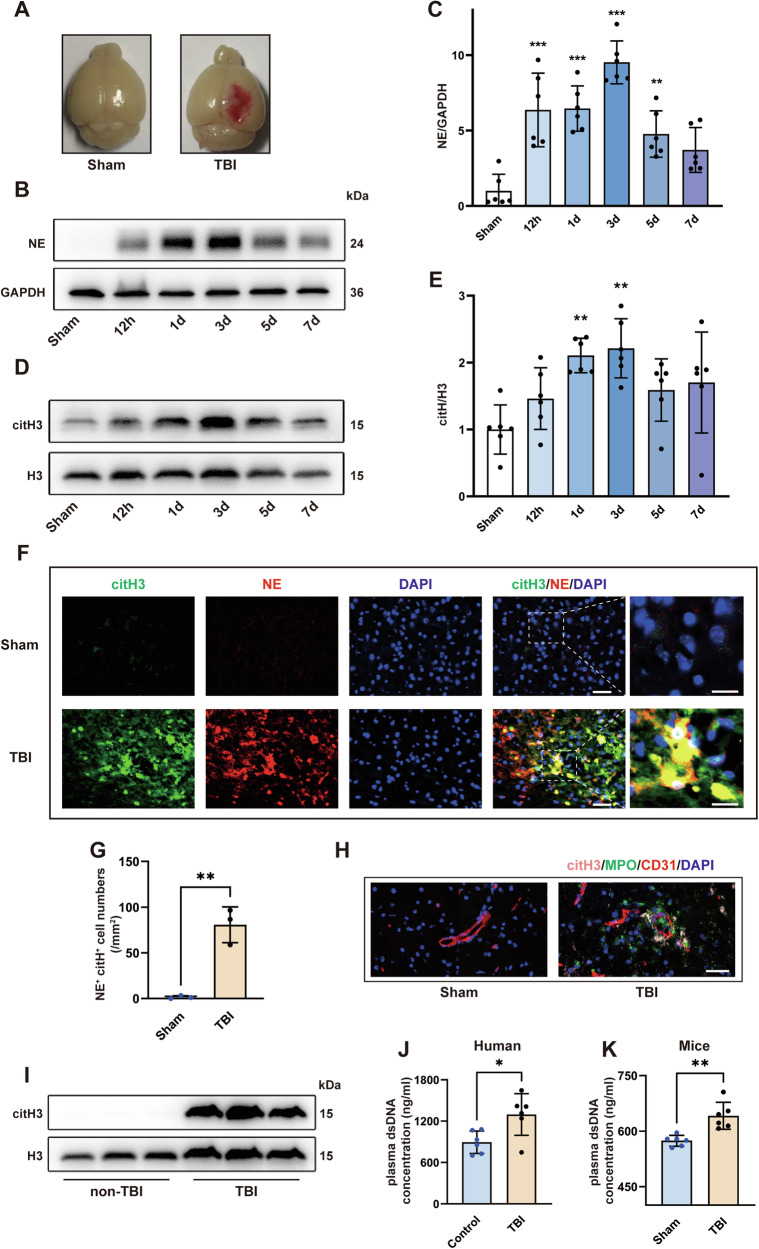
NETs are induced around cerebral vessels after TBI. **A** Significant foci of injury was present 3 days after TBI. **B**, **C** Western blotting and quantitative analysis of neutrophil-specific marker, NE, in the cortex (*n* = 6). **D**, **E** Western blotting and quantitative analysis of the NET-specific marker citH3 in the cortex (*n* = 6). **F**, **G** Representative image of double immunofluorescence staining of citH3 (green) and NE (red) and quantification of NE^+^ citH3^+^ cells in the cortex of mice from the sham and TBI groups 3 days after TBI (*n* = 3). Nuclei were stained with DAPI (blue). Scale bars = 50 μm, scale bars on the enlarged images = 10 μm. **H** Immunofluorescence staining of citH3 (pink), MPO (green), and CD31 (red). Nuclei were stained with DAPI (blue). Scale bar = 50 μm. **I** Western blotting of the NET-specific marker citH3 in the contused brain tissue of patients with and without TBI. **J** Plasma dsDNA concentrations of healthy donors and patients with TBI (*n* = 6). **K** Plasma dsDNA concentrations of the sham and TBI group (*n* = 6). Data were presented as the mean ± SD, ns: Not significant. *p < 0.05, **p < 0.01, ***p < 0.001.

Mice were subjected to TBI, which caused substantial loss of cortical tissue 3 days later, whereas that of sham mice was intact (Fig. 1A), indicating the successful establishment of Feeney’s WDI model. First, we collected brain tissue for western blot analysis at 12 h, 1 d, 3 d, 5 d, and 7 d after TBI. The neutrophil-specific marker NE was significantly elevated in the contused cortex after TBI, and reached a peak at 3 days after insult, indicating the presence of a large number of neutrophils in the ipsilateral cerebral cortex (Fig. 1B, C). The high expression of NE, a key molecule in the process of NET production [31], implied the generation of NETs. Thus, we further examined the expression of citrullinated histone H3 (citH3), a specific NET marker. The results were consistent with previous findings, demonstrating high levels of NETs in the contused cortex, peaking on day 3 after TBI (Fig. 1D, E). Therefore, we selected day 3 after TBI as the time point for indicator determination in the following experiments. Furthermore, because NETs contain multiple components, including NE and citH3, we detected the colocalization of NE, citH3, and DNA by immunofluorescence. Consistent with the western blot results, the NE and citH3 levels were significantly increased compared to that in the sham mice after TBI. Additionally, citH3^+^ neutrophils were readily found in the ipsilateral cerebral cortex (Fig. 1F, G), indicating that they were generating NETs or that NETs had already been assembled and released into the extracellular space. Because neutrophils originate from blood, we labeled the cerebral vessels using CD31. Immunofluorescence staining revealed that NETs produced by neutrophils were enriched around blood vessels (Fig. 1H). Next, to investigate whether NETs also exist in contused brain tissue from patients with TBI, we collected brain samples from patients with TBI for western blot analysis. Likewise, a significant elevation of citH3 was observed in the brain tissues of patients with TBI compared to those without (Fig. 1I and Fig. S1A, B). We also collected blood samples from TBI patients and healthy donors to measure the concentration of dsDNA in plasma. The results showed that the plasma concentration of dsDNA was significantly higher in patients with TBI than in healthy donors (*p* < 0.05, Student’s *t*-test, Fig. 1J). Similarly, the dsDNA concentrations in the plasma of TBI mice were also significantly increased compared with sham mice (Fig. 1K).

### NETs disrupt BBB integrity after TBI

**Fig. 2:**
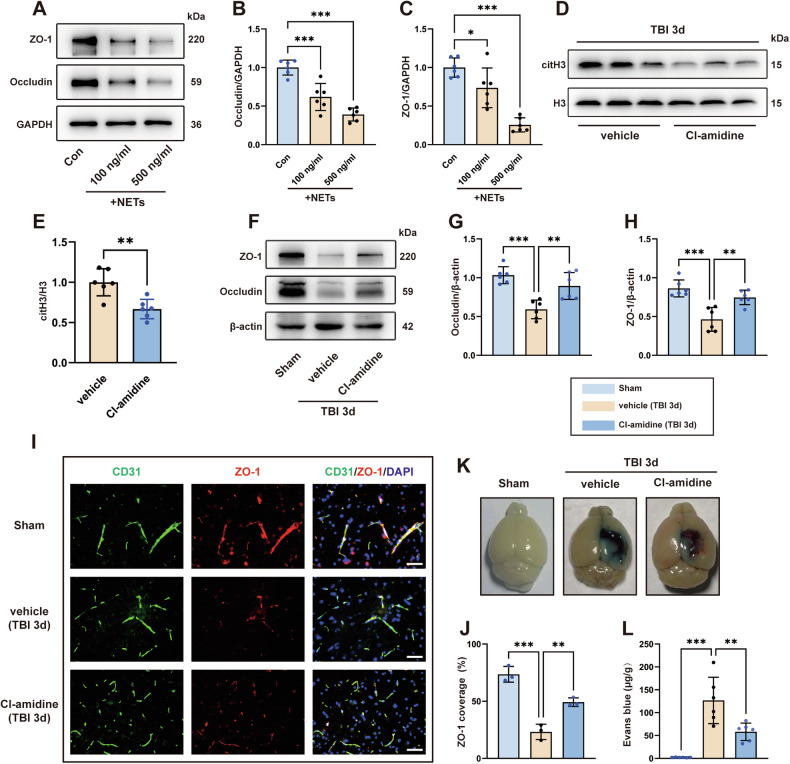
NETs disrupt BBB integrity after TBI. **A**–**C** Western blotting and quantitative analysis of tight junction proteins (ZO-1 and occludin) in hCMECs from control and different concentrations of NETs (100 ng/mL and 500 ng/mL for 24 h) stimulation groups (*n* = 6). **D**, **E** Western blotting and quantitative analysis of citH3 3 days after TBI (*n* = 6). **F**–**H** Western-blotting and quantitative analysis of tight junction proteins (ZO-1 and occludin) in the cortex 3 days after TBI. **I**, **J** Representative images of double immunofluorescence staining of CD31 (green) and ZO-1 (red), and quantification of the percentage of ZO-1-covered area (%CD31 area) in the cortex 3 days after TBI (*n* = 3). Nuclei were stained with DAPI (blue). Scale bars = 50 μm. **K** Representative images of brain tissue from the indicated treatment groups 3 days after TBI. The blue area indicates extravasation of Evans blue dye. **L** Quantification of leaked Evans blue dye in the ipsilateral cerebral hemisphere of mice from the indicated groups (n = 6). Data were presented as the mean ± SD, ns not significant. *p < 0.05, **p < 0.01, ***p < 0.001.

Previous studies have shown that NETs can cause endothelial cell dysfunction, leading to leakage of the vascular barrier [17, 24]. Therefore, we next investigated the effect of NETs on the function of the BBB in TBI. We first treated hCMECs with different concentrations of NETs in vitro. The results showed that the expression of occludin and ZO-1 decreased significantly after stimulation with 100 ng/mL NETs for 24 h, and the decrease was more pronounced when the concentration of NETs was increased to 500 ng/mL (Fig. 2A–C). To further investigate the effect of NETs on BBB function after TBI in vivo, we administered the PAD4 selective inhibitor Cl-amidine once per day for 3 consecutive days to inhibit the production of NETs after TBI. As shown in Fig. 2D, E, the citH3 level in the contused cortex after Cl-amidine administration was significantly reduced compared to that in the TBI+vehicle group, demonstrating that Cl-amidine effectively reduced the formation of NETs. Subsequently, the western blot results showed that the expression of ZO-1 and occludin decreased dramatically after TBI exposure in the TBI+vehicle group but that Cl-amidine post-treatment effectively rescued this decrease (Fig. 2F–H). Additionally, CD31-labeled blood vessels were co-stained with ZO-1, and the immunofluorescence staining results showed that ZO-1 covered most of the CD31^+^ regions in the sham group, whereas the expression of ZO-1 in blood vessels in the TBI+vehicle group was significantly reduced. However, Cl-amidine alleviated the loss of ZO-1 in bECs (Fig. 2I, J). To further observe the integrity of the BBB, Evans blue extravasation was performed 3 days after TBI. The results showed that because of the intact BBB, there was almost no Evans blue dye in the sham group, but substantial Evans blue leakage into the brain parenchyma was observed in mice subjected to TBI. However, BBB leakage was attenuated in mice treated with Cl-amidine (Fig. 2K, L). Overall, these results confirmed the important role of NETs in the development of BBB breakdown after TBI.

### NETs promote bEC pyroptosis via the TLR4/NF-κB pathway

**Fig. 3:**
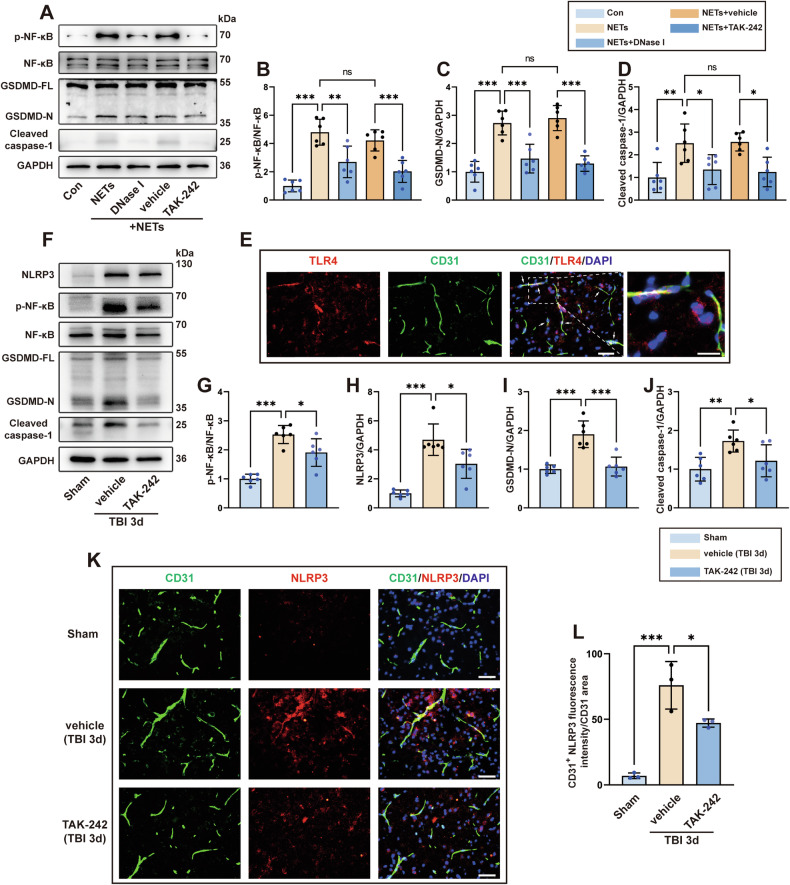
NETs promote bEC pyroptosis via the TLR4/NF–κB pathway. **A**–**D** Western blotting and quantification of the upstream of TLR4 (NF–κB and p-NF-κB) and pyroptosis-related proteins (GSDMD-N and cleaved caspase-1) in hCMECs (*n* = 6). NF-κB was used as the loading control for p-NF-κB quantification, and GAPDH was used as the loading control for quantification of pyroptosis-related proteins. **E** Representative image of double immunofluorescence staining of CD31 (green) and TLR4 (red) in the cortex. Nuclei were stained with DAPI (blue). The white asterisk indicates colocalization of CD31 and TLR4. Scale bar = 50 μm, scale bar on the enlarged images = 10 μm. **F**–**J** Western blotting and quantitative analysis of p-NF-κB and pyroptosis-related proteins (GSDMD-N, cleaved caspase-1, and NLRP3) in the cerebrovascular component of the cortex 3 days after TBI. NF-κB was used as the loading control for p-NF-κB quantification, and GAPDH was used as the loading control for the quantification of pyroptosis-related proteins. **K**, **L** Representative image of double immunofluorescence staining of CD31 (green) and NLRP3 (red) and quantification of NLRP3 expression in the cortex 3 days after TBI (*n* = 3). Nuclei were stained with DAPI (blue). Scale bars = 50 μm. Data were presented as the mean ± SD, ns not significant. *p < 0.05, **p < 0.01, ***p < 0.001.

Next, we investigated whether BBB destruction caused by NETs is related to bEC pyroptosis. As shown in Fig. 3A–D, NF-κB was highly phosphorylated after NET stimulation, and two important proteins, GSDMD and caspase-1 that mediate cell pyroptosis, were activated. These observations indicated that NETs can induce hCMEC pyroptosis. However, phosphorylated-NF-κB (p-NF-κB) levels were significantly reduced in hCMECs after treatment with TAK-242, a specific inhibitor of TLR4. Furthermore, cleaved GSDMD and caspase-1 levels were also decreased, revealing alleviation of NET-induced pyroptosis. Additionally, DNase I exerted an analogous effect on TAK-242 via the direct degradation of NETs. These results demonstrated that NETs can induce hCMEC pyroptosis through the TLR4/NF-κB pathway.

We next sought to determine whether the above process occurs in vivo following TBI. First, we demonstrated the expression of TLR4, caspase-1, GSDMD, and NLRP3 in bECs via immunofluorescence staining (Fig. 3E, K and Fig. S1D). Considering that pyroptosis occurs in multiple cell types in the CNS after TBI, we isolated the blood vessels of the contused cortex to extract proteins for western blot analysis. Compared to the sham group, p-NF-κB was significantly increased in the TBI+vehicle group. Meanwhile, NLRP3, GSDMD-N, and cleaved caspase-1 levels were also increased in the TBI+vehicle group compared to the sham group, indicating that the bECs underwent pyroptosis after TBI. However, the increase in p-NF-κB and pyroptosis-related proteins was reversed in mice intraperitoneally treated with TAK-242 (Fig. 3F–J). In addition, immunofluorescence staining showed that the expression of NLRP3 in bECs was dramatically increased in the TBI+vehicle group compared to the sham group. However, treatment with TAK-242 effectively reduced NLRP3 expression in bECs (Fig. 3K, L).

### NINJ1 is activated in pyroptotic bEC

**Fig. 4:**
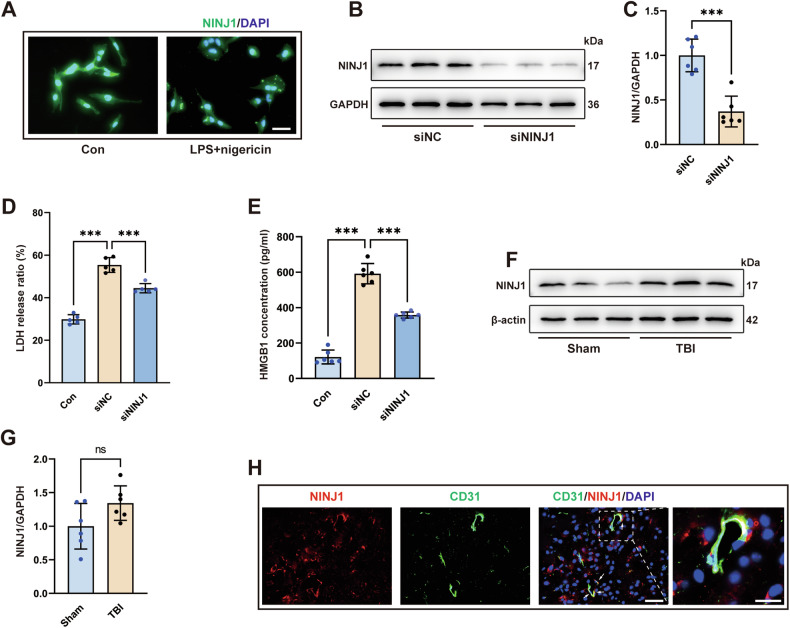
NINJ1 is activated in pyroptotic bEC. **A** Representative image of immunofluorescence staining of NINJ1 (green) in hCMECs. Nuclei were stained with DAPI (blue). The white asterisk indicates the cluster of NINJ1. Scale bars = 50 μm. **B**, **C** Western blotting and quantification of NINJ1 in hCMECs treated with siNC or siNINJ1 (*n* = 6). **D** Quantification of the release ratio of the PMR marker LDH in the supernatant medium of hCMECs, in which the siNC and siNINJ1 groups were both treated with LPS+nigericin (*n* = 5). **E** Quantification of the concentration of HMGB1 in the supernatant of hCMECs, in which the siNC and siNINJ1 groups were both treated with LPS+nigericin (*n* = 6). **F**, **G** Western blotting and quantification of NINJ1 in the cortex 3 days after TBI (*p* = 0.1486, *n* = 6). **H** Representative immunofluorescence staining of CD31 (green) and NINJ1 (red) in the cortex. Nuclei were stained with DAPI (blue). The white asterisk indicates colocalization of CD31 and NINJ1. Scale bar = 50 μm, scale bar on the enlarged images = 10 μm. Data were presented as the mean ± SD, ns not significant. *p < 0.05, **p < 0.01, ***p < 0.001.

As our results suggested that NETs induce bEC pyroptosis, we further investigated whether NINJ1 is involved in PMR in pyroptotic bECs. In vitro, we induced pyroptosis of hCMECs with LPS+nigericin. As shown in Fig. 4A, after pyroptosis induction, NINJ1 was clustered on the cell membrane, but it did not cluster in the control group. The observations revealed that NINJ1 in hCMECs was activated and oligomerized on the cell membrane after pyroptosis induction. To further investigate the function of NINJ in hCMECs, we designed three-stranded siRNAs to knock down NINJ (Fig. S1E). The results showed that NINJ1 was knocked down successfully (Fig. 4B, C). Next, we detected the release of LDH, which is a marker of PMR. Specifically, the siNINJ1 and siNC groups received pyroptosis induction, while the control group was not subjected to any treatment. Interestingly, although the siNC and siNINJ1 groups received the same LPS+nigericin treatment, LDH release was significantly reduced after knocking down NINJ1 (Fig. 4D). Subsequently, we detected the release of HMGB1 in the cell supernatant. The results showed that the release of HMGB1 was also reduced in the siNINJ1 group compared to the siNC group after pyroptosis induction (Fig. 4E).

Next, NINJ1 expression in the brain was analyzed by immunofluorescence staining. The results revealed that NINJ1 was expressed by bECs (Fig. 4H), whereas it was almost not expressed by astrocytes and microglia, and there was weak colocalization between neurons and NINJ1 (Fig. S1F). Moreover, double immunofluorescence of NINJ1 and GSDMD was performed (Fig. S1G). Following TBI, NINJ1 expression was found to be increased (*p* = 0.1486, Fig. 4F, G). Taken together, these results suggest that NINJ1 plays an important role in the amplification of inflammation after TBI.

### NINJ1 knockdown mitigates neutrophil infiltration and NET formation after TBI

**Fig. 5:**
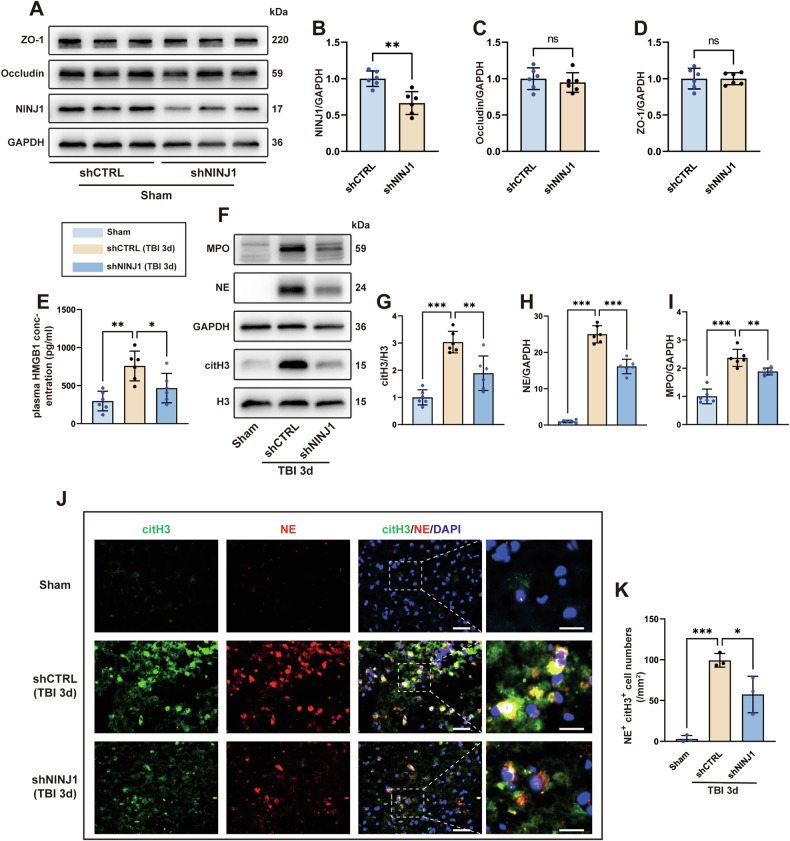
Knockdown of NINJ1 mitigates neutrophil infiltration and NET formation. **A**–**D** Western blotting and quantification of NINJ1 and TJPs (ZO-1 and occludin) in the cortex of mice treated with AAV-TIE-shNC or AAV-TIE-shNINJ1 (*n* = 6). **E** Quantification of the concentration of HMGB1 in the plasma 3 days after TBI (*n* = 6). **F**–**I** Western blotting and quantification of the neutrophil (MPO and NE) and NET-specific (citH3) markers in the cortex 3 days after TBI (*n* = 6). **J**, **K** Representative image of immunofluorescence staining of citH3 (green) and NE (red) and quantification of NE^+^ citH3^+^ cells in the cortex 3 days after TBI. Nuclei were stained with DAPI (blue). Scale bar = 50 μm, scale bar on the enlarged images = 10 μm. Data were presented as the mean ± SD, ns not significant. *p < 0.05, **p < 0.01, ***p < 0.001.

To investigate the role of NINJ1 in bECs after TBI, mice were randomly divided into three groups: sham, TBI+shCTRL, and TBI+shNINJ1. AAV was administered into the lateral ventricle 21 days before TBI. First, to verify the knockdown efficiency, we analyzed NINJ1 expression in the cortical brain tissue before modeling. The results showed that AAV-TIE-shNINJ1 successfully decreased the expression of NINJ1 (Fig. 5A, B). Additionally, considering that AAV was designed to target endothelial cells, the expression of tight junction proteins (ZO-1 and occludin) was detected, and the western blot bands indicated that the cerebrovascular barrier was not affected after knocking down NINJ1 in mice without TBI (Fig. 5A, C, D). We had confirmed that the release of HMGB1 from pyroptotic hCMECs was reduced after knockdown of NINJ1, because bECs also underwent pyroptosis after TBI, we determined the concentration of HMGB1 in mouse plasma. The ELISA results showed that the plasma HMGB1 concentration of mice in the TBI+shCTRL group was significantly higher than that in the sham group, whereas this increase was reversed in the TBI+shNINJ1 group (Fig. 5E). As numerous studies have demonstrated that HMGB1 can effectually promote NET formation, we next analyzed the neutrophil and NET levels after TBI. The results showed that the neutrophil markers MPO and NE and the NET-specific marker citH3 were all significantly decreased in the TBI+shNINJ1 group compared to the TBI+shCTRL group (Fig. 5F–I). Meanwhile, double immunofluorescence staining was further supported the western blot results, which was characterized by a decrease in NE^+^ citH3^+^ cell numbers compared to the TBI+shCTRL group (Fig. 5J, K).

In summary, by knocking down NINJ1 in bECs, the release of HMGB1 was reduced, which alleviated neutrophil infiltration and NET formation following TBI.

### NINJ1 knockdown alleviates NET-mediated bEC pyroptosis after TBI

**Fig. 6:**
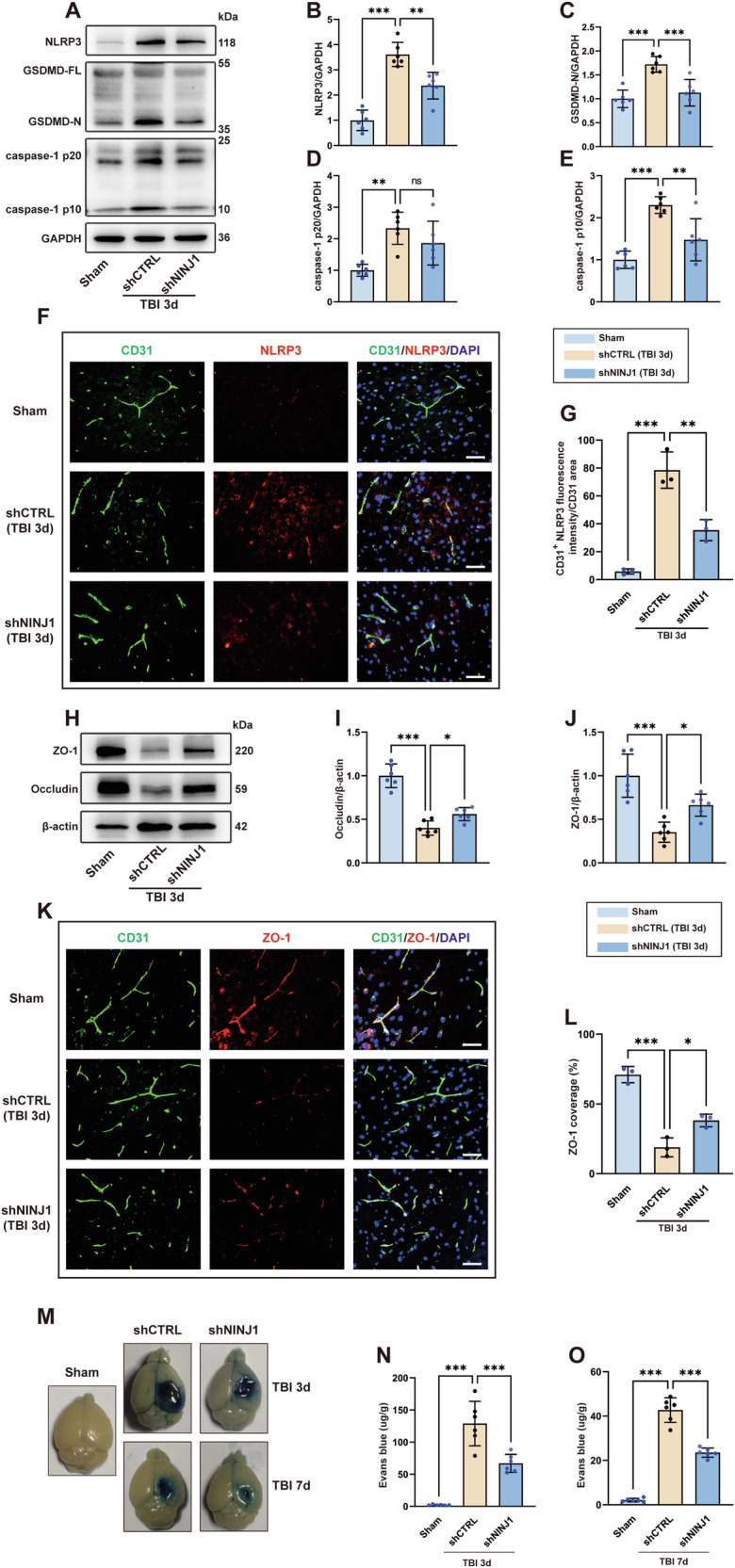
Knockdown of NINJ1 ameliorates BBB destruction by alleviating bEC pyroptosis after TBI. **A**–**E** Western-blotting and quantification of pyroptosis-related proteins (GSDMD-N, caspase-1 p20, caspase-1 p10, and NLRP3) in the cerebrovascular component of the cortex 3 days after TBI (**D**, *p* = 0.2771) (*n* = 6). **F**, **G** Representative image of immunofluorescence staining of CD31 (green) and NLRP3 (red) and quantification of the expression of NLRP3 in the cortex 3 days after TBI (*n* = 3). Nuclei were stained with DAPI (blue). Scale bar = 50 μm. **H**–**J** Western blotting and quantification of tight junction proteins (ZO-1 and occludin) in the cortex 3 days after TBI (*n* = 6). **K**, **L** Representative image of double immunofluorescence staining of CD31 (green) and ZO-1 (red) and quantification of the percentage of ZO-1-covered area (%CD31 area) in the cortex 3 days after TBI (*n* = 3). Nuclei were stained with DAPI (blue). Scale bar = 50 μm. **M** Representative images of brain tissue from the indicated treatment groups 3 or 7 days after TBI. The blue area indicates extravasation of Evans blue dye. **N**, **O** Quantification of leaked Evans blue dye in the ipsilateral cerebral hemisphere of mice from the indicated groups (*n* = 6). Data were presented as the mean ± SD, ns not significant. *p < 0.05, **p < 0.01, ***p < 0.001.

As we confirmed that neutrophils and NETs in the contused cortex can be reduced by knocking down NINJ1, we next sought to explore whether knocking down NINJ1 could reduce BBB destruction by alleviating bEC pyroptosis after TBI. As shown in Fig. 6A–E, when compared to the mice in the TBI+shCTRL group, the expression of the pyroptosis-related proteins NLRP3, caspase-1 p20, caspase-1 p10, and GSDMD-N in bECs was downregulated in mice receiving AAV-TIE-shNINJ1. Consistent with the western blot results, immunofluorescence staining showed that the expression of NLRP3 in bECs in the TBI+shNINJ1 group was remarkably decreased compared to that in the TBI+shCTRL group (Fig. 6F, G).

### NINJ1 knockdown reduces BBB destruction after TBI

The expression of ZO-1 and occludin was analyzed by western blotting, and the results indicated that the decreasing trend of ZO-1 and occludin expression after TBI was effectively rescued in mice with NINJ1 knockdown (Fig. 6H–J). Similarly, double immunofluorescence staining of blood vessels and ZO-1 further supported western blotting, as reflected by the increased percentage of blood vessels covered by ZO-1 (Fig. 6K, L). Evans blue was intravenously injected into mice 3 and 7 days after TBI. Compared to the TBI+shCTRL group, the extent of Evans blue leakage into the brain parenchyma was less in the TBI + AAV-shNINJ1 group at 3 days post-injury. Additionally, although the total amount of Evans blue dye that leaked into the brain parenchyma was decreased at 7 days post-injury, the amount of Evans blue dye in the brain parenchyma was still lower in the TBI+shNINJ1 group than in the TBI+shCTRL group (Fig. 6M–O).

### Knockdown of NINJ1 improves neurological outcomes after TBI

**Fig. 7:**
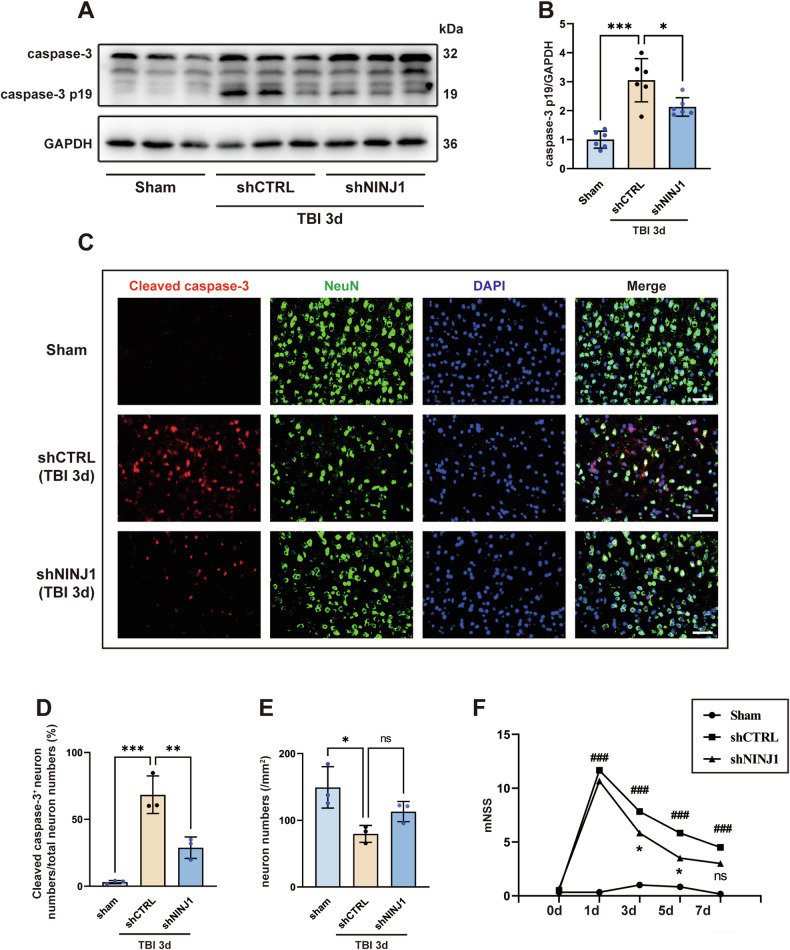
Knockdown of NINJ1 improves neurological outcomes after TBI. **A**, **B** Western blotting and quantification of apoptosis-related protein caspase-3 p19 in the cortex 3 days after TBI (*n* = 6). **C**, **D** Representative image of immunofluorescence staining of NeuN (green) and cleaved caspase-3 (red) and quantification of cleaved caspase-3-postive neurons cells in the cortex 3 days after TBI (*n* = 3). Nuclei were stained with DAPI (blue). Scale bar = 50 μm. **E** Quantification of neurons in the cortex 3 days after TBI (*P* = 0.2128, *n* = 3). **F** mNSS neurological function scores were assessed 1 day to 1 week after TBI in different groups of mice (*n* = 6). Data were presented as the mean ± SD, ns not significant, when the TBI + AAV-shCTRL group was compared to the sham group, ###*p* < 0.001, when the TBI + AAV-shNINJ1 group was compared to the TBI + AAV-shCTRL group, *p < 0.05, **p < 0.01, ***p < 0.001.

The programmed cell death pathway of neurons triggered by neurotoxic proteins in the blood is an important factor for neuronal loss and is a major contributor to motor and cognitive impairments following TBI [32]. Consequently, the expression of cleaved caspase-3 was analyzed to detect neuronal apoptosis, which showed that caspase-3 p19 was upregulated in the TBI+shCTRL group when compared to the sham group, whereas the upregulation in mice receiving AAV-TIE-shNINJ1 was reversed (Fig. 7A, B). Double immunofluorescence staining further supported the western blot results. Specifically, the percentage of apoptotic neurons was reduced after NINJ1 knockdown in the TBI+shNINJ1 group compared to the TBI+shCTRL group (Fig. 7C, D). We also counted the total number of neurons per field in the cortex. Compared to the sham mice, the number of neurons was markedly reduced within the contused cortex of the mice in the TBI+shCTRL group, whereas the TBI+shNINJ1 group exhibited a tendency of alleviated neuron loss (*p* = 0.2128, Fig. 7E). Next, the mNSS was investigated by two researchers who were blinded to the experimental design. As shown in Fig. 7F, the neurological deficits of mice in the TBI+shNINJ1 group were significantly improved compared to mice in the TBI+shCTRL group.

## Discussion

**Fig. 8:**
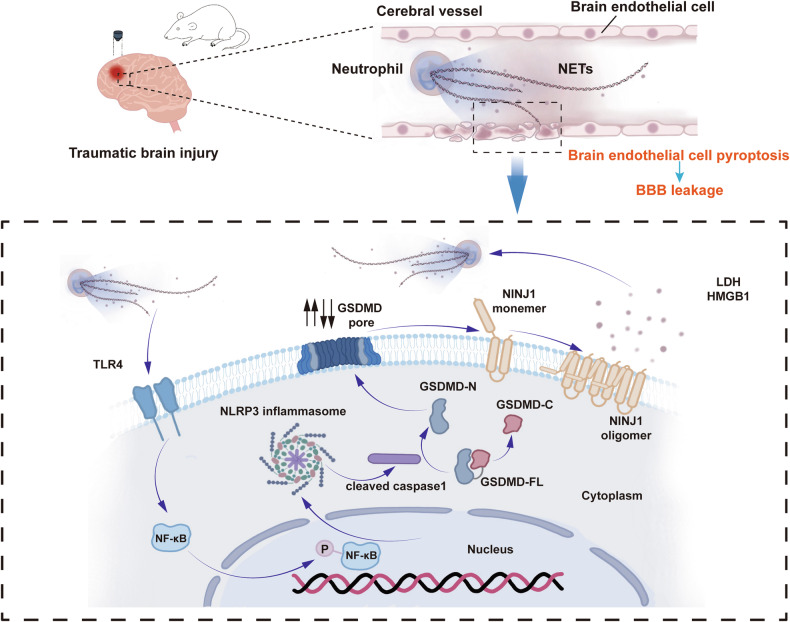
NINJ1-mediated plasma membrane rupture of pyroptotic endothelial cells exacerbates blood-brain barrier destruction caused by neutrophil extracellular traps in traumatic brain injury. NETs induced by TBI interact with the TLR4 of bECs and then activate NF-κB to transfer into the nucleus, which promotes NLRP3 inflammasome formation. Then, activated caspase-1 from the NLRP3 inflammasome cleaved GSDMD and released GSDMD-N, perforating the GSDMD pores and increasing the BBB permeability. Furthermore, GSDMD pores activate NINJ1 oligomerization on the cell membrane, which mediates PMR. HMGB1 promotes neutrophil formation in NETs.

BBB disruption is an early event that may persist for a long time following TBI, and the secondary injury cascade is a significant factor in persistent BBB dysfunction [33]. At the onset of TBI, the vascular barrier is directly damaged by mechanical forces, which allow massive peripheral immune cells to enter the brain parenchyma and release related cytokines and reactive oxygen species, leading to neuronal damage [3, 4]. Furthermore, damaged cells release cellular contents, which are identified by immune cells as DAMPs, leading to the expansion of the inflammatory response [5]. Meanwhile, long-term neuroinflammation leads to sustained damage to the BBB. BECs are in direct contact with the bloodstream, and play a crucial role in the regulation of inflammation [34]. A recent study revealed that bECs are the first cell type in the CNS to respond to LPS-induced inflammation and exhibit the most profound change, characterized by the significant upregulation of signature genes mediating the inflammatory and immune responses [22]. Neutrophils are a major arm of the innate immune system and are found to be the first peripheral immune cells recruited following TBI [11, 35]. Various interaction mechanisms between bECs and neutrophils have been reported following TBI. Here, we reported a novel crosstalk between bECs and neutrophils in which NINJ1-mediated PMR of bECs aggravated NET-induced bEC dysfunction (Fig. 8).

Previous studies have confirmed that NETs are present in the lesion tissues of patients with stroke and TBI and are associated with the exacerbation of neurological deficits [36, 37]. Similarly, we confirmed that NETs were present in the contused tissue of patients with TBI via western blot analysis of citH3. Meanwhile, elevated NETs in the peripheral blood circulation of TBI patients and mice were further verified by dsDNA quantification. Circulating DNA in blood had been widely regarded as a DAMP in recent research. Cell-free DNA (cf-DNA), mainly originating from NETosis, was revealed that could activate absent in melanoma 2 (AIM2) inflammasome, contributing to the high recurrence rate after stroke [38]. Moreover, dsDNA in NETs could be detected by corresponding DNA sensors, such as toll-like receptor 9 (TLR9) [39], and could initiate the cyclic GMP–AMP synthase (cGAS)/stimulator of interferon genes (STING) pathway to aggravate inflammation [17, 40]. In addition to trapping pathogens, NETs are associated with multiple injuries, among which vascular injury is an important type of damage induced by NETs. Correspondingly, NETs can disrupt the BBB and impair revascularization in the CNS [41]. Our study showed that neutrophils and NETs were enriched around blood vessels after TBI, suggesting that they play an important role in BBB dysfunction. Cl-amidine is a potent oral PAD4 inhibitor, which can block the citrullination of H3 and thus inhibits the formation of NETs, it can effectively alleviate various types of damage caused by NETs [17, 36, 42]. Consistent with previous studies, the decline in thigh junction proteins and extravasation of Evans blue were rescued by Cl-amidine treatment, which verified the destructive effect of NETs on BBB breakdown following TBI.

It has been reported that NETs can lead to endothelial dysfunction and induce pyroptosis in various cell types in different diseases [43–45]. In this study, we used NETs to stimulate hCMECs and found increased expression of caspase-1 p20, which could cleave full-length GSDMD (GSDMD-FL) to release activated N-terminal GSDMD (GSDMD-N). Correspondingly, the level of GSDMD-N was also increased after NET stimulation. The above results indicated that NET-induced BBB disruption was related to bEC pyroptosis. We also explored how NETs led to bEC pyroptosis. It is widely accepted that the occurrence of pyroptosis is often related to the activation of innate immune-related pathways. TLR4 plays an important recognition role in the innate immune system [46]. Importantly, NE had been reported to interact with TLR4 to initiate the TLR4/NF-κB pathway, which was significantly elevated following TBI [47]. Thus, we examined the activation of NF-κB, downstream of TLR4, which promoted the transcription of inflammatory factors and induced cell pyroptosis. We found that p-NF-κB was significantly increased in NET-treated hCMECs, whereas the activation of NF-κB was rescued by blocking TLR4, confirming that NETs could indeed be recognized by TLR4. Moreover, the pyroptosis of hCMECs was effectively rescued by TAK-242 treatment. Consistently, our findings in an experimental TBI revealed that bEC pyroptosis was attenuated by TAK-242 administration after TBI. Taken together, these findings revealed a novel mechanism of BBB breakdown caused by NETs.

As an inflammatory form of programmed cell death, pyroptosis leads to inflammatory factors released via PMR. PMR was previously thought to be a passive process resulting from osmotic pressure changes. However, recent studies indicated that PMR is mediated by the activation of NINJ1 and that oligomerized NINJ1 forms filamentous assemblies with branched morphology for cutting the plasma membrane [48–50]. Previous investigations had shown that NINJ1 is expressed by bECs in the CNS [51, 52], which was also confirmed in our study. Thus, we speculated that NINJ1 also mediates PMR in pyroptotic bECs during TBI. Indeed, the release ratio of the PMR maker LDH and HMGB1 in the cell supernatant were significantly decreased after knocking down NINJ1 in hCMECs following the induction of pyroptosis. Notably, NINJ1 is activated under pathological conditions, which may suggest that NINJ1 is a promising target for intervention without affecting normal physiological function.

HMGB1, a structural protein of chromatin that can be actively secreted into the extracellular space by cells or passively released from damaged cells, is a key mediator of sterile inflammation and NET formation [19, 30, 37, 53]. Additionally, HMGB1 is a relatively small nuclear protein (~28 kDa in size), but it forms large complexes with nucleosomes and transcription factors, which likely hinders its release through the approximately 18-nm GSDMD pore [26, 54]. Therefore, we hypothesized that NET accumulation could be rescued by intervening NINJ1 in bECs after TBI. As expected, neutrophil infiltration and NETs were decreased after NINJ1 knockdown, which effectively attenuated NET-induced bEC dysfunction after TBI. Moreover, the extent of BBB disruption was reduced and neurological function was improved after TBI.

This study has some limitations that warrant discussion. We demonstrated that NETs can induce bEC pyroptosis via the TLR4/NF-κB pathway, thereby leading to BBB leakage. However, other underlying mechanisms for NET-related bEC dysfunction following TBI, such as oxidative stress, calcium overload, and other cell death forms, cannot be disregarded. Furthermore, in addition to mediating PMR in pyroptotic cells, NINJ1 exerts other functions in living cells, including angiogenesis and bone homeostasis, and acts as a target of p53 to regulate cell death [55–57]. Therefore, it will be important to conduct a prospective investigation to corroborate our findings.

In conclusion, our investigation demonstrated the key role of NETs in BBB breakdown after TBI and revealed the underlying mechanism, i.e., NETs activated the TLR4/NF-κB pathway of bECs and then initiated the canonical caspase-1/GSDMD pyroptosis pathway to induce bEC pyroptosis, which increased BBB permeability. Subsequently, HMGB1 was released from pyroptotic bECs via NINJ1-mediated PMR and promoted neutrophils to form more NETs. We interrupted the vicious cycle by knocking down NINJ1 in bECs and successfully alleviated BBB disruption in the acute phase of TBI. Therefore, we identified NINJ1 as a novel drug design target for NET-induced BBB destruction and other pathological processes associated with NETs after TBI.

## Material and methods

### Human samples

Human brain tissue and blood were collected using a protocol approved by the ethics committee of Jinling Hospital (approval No. 2024DZGJJ-190). Brain tissues were obtained from patients with severe TBI and those with glioma who underwent maximum resection of malignant glioma to improve survival. For the blood sample, 2 mL of blood sample was collected from healthy donors or patients within 1 week of TBI onset. Informed consent was obtained from all participants. The clinical characteristics of all patients and healthy donors are shown in Supplementary Table.

### Animals

Male C57BL/6 mice (6–8 weeks old, 20–25 g) were purchased from the Model Animal Research Center of Nanjing University (Nanjing, China). Animals were housed under constant temperature (26 ± 2°C) and humidity (50 ± 10%) under a 12-h light/12-h dark circle. All animals had unrestricted access to water and food.

All procedures involving animals were performed in accordance with the Guidelines for the Care and Use of Laboratory Animals published by the National Institutes of Health and approved by the Animal Care and Use Committee of Jinling Hospital (approval No. DZGZRDW2400228).

### Weight-drop injury model

We used a modified Feeney’s weight-drop injury model, as previously described [58]. In brief, mice were anesthetized with 2% isoflurane and then maintained with 1% isoflurane. First, we used a scalpel to expose the skull, before placing the weight-drop device 0.8 mm posterior to the bregma and 1.3 mm lateral to the sagittal suture in the right hemisphere. Next, TBI was induced using a 2-mm impacting rod, and a 100-g weight was dropped from a height of 5 cm. After TBI, the mice were placed on a heating pad. The sham group underwent all of the same operations except for the strike.

### In vivo drug administration

The PAD4 selective inhibitor Cl-amidine (GC35706, GLPBIO, Montclair, CA, USA) was dissolved in dimethyl sulfoxide (DMSO) and diluted with sterile saline (5% v/v) at a concentration of 4 mg/mL. Cl-amidine was intraperitoneally injected into mice at a dose of 50 mg/kg at 10 min after TBI and then once a day for 3 consecutive days. The TLR4 selective inhibitor TAK-242 (HY-11109, MedChemExpress, Shanghai, China) was dissolved in DMSO and diluted with sterile saline (5% v/v) at a concentration of 0.5 mg/mL. TAK-242 was delivered (3 mg/kg, intraperitoneally) 30 min after TBI and then once a day for 3 consecutive days [59]. The vehicle group delivered the same volume of 95% saline and 5% DMSO mixture once daily.

### Adeno-associated virus (AAV) injection

To knock down NINJ1 in bECs in vivo, adeno-associated virus AAV-TIE-shNINJ1 (1.20 × E13 v. g/mL) or empty control virus AAV-TIE-shNC (1.15 × E13 v. g/mL) (Genomeditech, Shanghai, China) was administered via intracerebroventricular injection. Briefly, 2% isoflurane was administered to mice as anesthesia, and the injection site was exposed (0.8 mm posterior and 1.2 mm lateral of the bregma, with an injection depth of 2.5 mm). During injection, mice were anesthetized with 1% isoflurane, and the virus was injected with a 10-μL microsyringe at a rate of 1 μL/min (4 μL total volume). After injection, the needle remained in the brain for 5 min to prevent liquid reflux.

### Cell culture

Human cerebral microvascular endothelial cells (hCMEC/D3) were purchased from Zhong Qiao Xin Zhou Biotechnology (ZQ0961, Shanghai, China). HCMECs were cultured with Prigrow I Medium (TM001, Applied Biological Materials, Vancouver, Canada) containing 10% fetal bovine serum (FBS) (10099141, Gibco, Grand Island, NY, USA) and 1% penicillin-streptomycin (151140122, Gibco) in an incubator with a humidified atmosphere of 95% air and 5% CO_2_ at 37 °C. The medium was changed two to three times per week.

### Small interfering RNA (siRNA) transfection

The siRNA targeting human *Ninj1* was purchased from Shanghai Hanbio Biotechnology (siNINJ1: AUGAAGAUGUUGACUACCACG, siNC: ACGUGACACGUUCGGAGAA). The siRNA transfection was performed according to the manufacturer’s instructions using jetPRIME (101000046, Polyplus, Strasbourg, France). The medium was changed 6 h after transfection, and the hCMECs were cultured for 48 h for future assays.

### Drug treatment and induction of pyroptosis in vitro

The medium was changed once before treatment. TAK-242 (30 μM) was administered 30 min before NETs (100 ng/mL) stimulation to pre-block TLR4. DNase I (D7073, Beyotime, Shanghai, China) was used to degrade NETs. NETs were first incubated with DNase I at 37 °C for 30 min and then added to 6-well plates. HCMECs were co-cultured with NETs for 24 h for western blot analysis.

HCMECs were first primed with 10 μg/mL lipopolysaccharide (LPS) (HY-D1056, MedChemExpress, Shanghai) for 4 h, and then pyroptosis was induced by 20 μM nigericin (T3092, TargetMol, Shanghai, China) for 30 min.

### Quantification of plasma dsDNA

The mouse whole blood from the heart was collected in a 1.5-ml centrifuge tube containing EDTA-Na, and the plasma was acquired by centrifugation at 4 °C, 1000×*g* for 20 min. Next, the dsDNA concentration in the plasma was quantified according to the manufacturer’s instructions using a dsDNA HS Assay Kit (12640ES60, Yeasen, Shanghai, China).

### Lactate dehydrogenase (LDH) assay

The cell supernatant was collected and then centrifuged at 4 °C, 500×*g* for 5 min to remove cell debris. The LDH release ratio was determined according to the manufacturer’s instructions for an LDH cytotoxicity detection kit (C0016, Beyotime, Shanghai). Subsequently, the absorbance of the sample was measured at 490 nm. The calculation method was based on the following formula:

$${LDH\; release\; ratio}\,\left(100 \% \right)=\frac{{A}_{{sample}}-{A}_{{blank\; control}}}{{A}_{\max }-{A}_{{balnk\; control}}}\times 100 \%$$

### HMGB1 enzyme-linked immunosorbent assay (ELISA)

The sandwich ELISA principle was used to determine the HMGB1 concentration. The specific procedures for detecting the HMGB1 concentration in mouse plasma were performed using a Mouse HMGB1 ELISA Kit (E-EL-M0676, Elabscience, Wuhan, China) according to the manufacturer’s instructions. For the cell supernatant samples, the culture medium was collected in centrifuge tubes, and the cell debris was removed via centrifugation at 4 °C, 1000×*g* for 20 min. The supernatant was then collected to detect the HMGB1 concentration using a Human HMGB1 ELISA Kit (E-EL-H1554, Elabscience, Wuhan) according to the manufacturer’s instructions.

### Neutrophil isolation

The peripheral blood of healthy donors was collected in a 2-mL anticoagulant tube. Neutrophil isolation was completed within 2 h so as to maintain the activity of neutrophils. In our investigation, gradient centrifugation was conducted to isolate neutrophils in blood using a Human Peripheral Blood Neutrophil Isolation Kit (P9040, Solarbio, Beijing, China) according to the kit instructions.

### NET isolation and quantification

Fresh neutrophils isolated from blood were seeded in six-well culture plates (3 × 10^6^ cells/well) and cultured with RPMI 1640 medium (11875093, Gibco) containing 10% FBS and 1% penicillin-streptomycin. Then, the cells were stimulated with 100 nM Phorbol 12-myristate 13-acetate (HY-18739, MedChemExpress, Shanghai) at 37 °C for 4 h. After stimulation, the medium was discarded and the six-well plates were gently washed with 1 mL of phosphate-buffered saline, before vigorously rinsing the bottom of the plates with Prigrow I Medium to obtain NETs. Next, the medium was collected for centrifugation at 500×*g* for 10 min to eliminate the cell debris. Finally, we collected the supernatant and stored it at –80 °C. The isolated NETs were quantified using a dsDNA HS Assay Kit.

### Brain vasculature isolation

The cerebral vessels of brain tissue were separated following the previous method [60]. Briefly, the brain tissue was homogenized with 3 mL cold sucrose buffer (0.32 M sucrose, 5 mM HEPES, pH = 7.4) using a Dounce homogenizer, and the homogenate was then centrifuged at 4 °C and 1000×*g* for 10 min. After centrifugation, we discarded the supernatant, resuspended the remaining pellet with 3 mL cold sucrose buffer, and subjected the sample to centrifugation at 2–8 °C, 350×*g* for 10 min four times. The final pellet contained the cerebrovascular enrichment components, which were lysed using radio-immunoprecipitation (RIPA) lysis buffer (P0013B, Beyotime, Shanghai) containing 1% phenylmethanesulfonyl fluoride (PMSF) (ST506, Beyotime, Shanghai) and 1% phosphatase inhibitor cocktail 2 (P5726, Sigma-Aldrich, City of Saint Louis, MO, USA) to extract proteins for western blotting.

### Western blot

The anesthetized mice were perfused with cold saline, before harvesting the brain tissue and storing at −80 °C. Proteins from the tissue or cells were extracted using RIPA lysis buffer containing 1% PMSF and 1% phosphatase inhibitor cocktail 2. The proteins were denatured at 95 °C for 10 min. Next, the proteins were separated via sodium dodecyl sulfate-polyacrylamide gel electrophoresis and transferred onto polyvinylidene difluoride membranes (IPVH00010, Millipore, Temecula, CA, USA). The transferred membranes were blocked with 5% skimmed milk at room temperature for 2 h and then incubated with the following primary antibodies at 4 °C overnight: anti-GAPDH (1:2000, 2118 s, Cell Signaling Technology, Danvers, MA, USA), anti-citH3 (1:1000, ab281584, Abcam, Cambridge, UK), anti-NE (1:400, sc-55549, Santa Cruz Biotechnology, Dallas, TX, USA), anti-MPO (1:1000, 22225-1-AP, Proteintech, China), anti-H3 (1:3000, 17168-1-AP, Proteintech), anti-β-actin (1:2000, 66009-1-Ig, Proteintech), anti-ZO-1 (1:2000, 21773-1-AP, Proteintech), anti-occludin (1:2000, 27260-1-AP, Proteintech), anti-NLRP3 (1:1000, 68102-1-Ig, Proteintech), anti-p-NF-κB (1:1000, 3033 s, Cell Signaling Technology), anti-NF-κB (1:1000, 8242 s, Cell Signaling Technology), anti-GSDMD (1:1000, ab209845, Abcam), anti-GSDMD (1:1000, A24476, Abclonal, China), anti-caspase-1 (1:1000, 22915-1-AP, Proteintech), anti-NINJ1 (1:300, sc-136295, Santa Cruz Biotechnology), and anti-caspase-3 (1:1000, 19677-1-AP, Proteintech). Following incubation, the membranes were washed with tris-buffered saline with Tween 20 and then incubated with anti-rabbit or anti-mouse IgG HRP-linked antibodies (1:5000, 7074 s, Cell Signaling Technology or 1:5000, 7076 s, Cell Signaling Technology) at room temperature for 90 min. Finally, an enhanced chemiluminescence system was used to visualize the protein bands. For different proteins with conflicting molecular weights, we use Western Blot Stripping Buffer (21059, Thermo Fisher Scientific, Waltham, MA, USA) to ensure incubation on the same membrane

### Immunofluorescence staining

After perfusion, the mouse brain was fixed in 4% paraformaldehyde at 4 °C for 12 h and dehydrated in 15 and 30% sucrose. The brains were sliced into 10-μm-thick coronal sections. The sections were first permeabilized with 0.1% Triton X-100 (P0096, Beyotime, Shanghai) for 15 min and blocked with normal goat serum for 30 min at room temperature. Subsequently, the sections were incubated with the following primary antibodies overnight at 4 °C: anti-citH3 (1:200, ab281584, Abcam), anti-NE (1:100, sc-55549, Santa Cruz Biotechnology), anti-CD31 (1:100, 550274, BD Pharmingen, Bergen County, NJ, USA), anti-MPO (1:200, 22225-1-AP, Proteintech), anti-NLRP3 (1:200, A24294, Abclonal), anti-NINJ1 (1:200, A24739, Abclonal), anti-NINJ1 (1:100, sc-136295, Santa Cruz Biotechnology), anti-ZO-1 (1:200, 21773-1-AP, Proteintech), anti-caspase-1 (1:200, 22915-1-AP, Proteintech), anti-GSDMD (1:200, A24476, Abclonal), anti-TLR4 (1:200, A5258, Abclonal), anti-neuronal nuclear protein (NeuN) (1:200, MAB3777X, MilliporeSigma, Billerica, MA, USA), anti-glial fibrillary acidic protein (GFAP) (1:200, 53-9892-82, Invitrogen, Carlsbad, CA, USA), anti-Iba-1 (1:200, GB15105, Servicebio, Wuhan, China), and anti-cleaved caspase-3 (1:200, 9661 s, Cell Signaling Technology). The sections were then incubated with the corresponding species’ distinctive fluorescently labeled antibodies (1:200, SA00009-2, Proteintech or 1:200, RGAM002, Proteintech) at room temperature for 90 min. Finally, 4ʹ,6- diamidino-2-phenylidole (DAPI, C1005, Beyotime, Shanghai) was used for nuclear staining, before observing the sections under a fluorescence microscope.

### Evans blue extravasation

To detect the BBB integrity, Evans blue solution (3 mL/kg, 4% in saline, E104208, Aladdin, Shanghai, China) was injected into mice via the tail vein. After 2 h of circulation, mice were anesthetized with 2% isoflurane and perfused with sterile saline. The brain tissue was then homogenized in 1 mL formamide and incubated at 50 °C for 48 h to extract Evans blue dye. After incubation, the supernatant was acquired via centrifugation, and the Evans blue concentration was detected at 620 nm using a Multiskan SkyHigh Microplate Spectrophotometer (Thermo Fisher Scientific, Waltham, MA, USA).

### Neurological function assessment

The modified neurological severity score (mNSS) was used to assess the neurological function of mice, which includes a composite of motor (muscle status and abnormal movement), sensory (visual, tactile and proprioceptive), reflex, and balance tests. The mice were examined once before TBI and then 1, 3, 5, and 7 days after TBI. Each point was given when the mice were unable to perform one of the tests. The total possible mNSS score was 18 (0, normal; 1–6, mild; 7–12, moderate; 13–18, severe).

### Statistical analysis

All data analyses were performed using GraphPad Prism 8.0, and the values for each group are presented as the mean ± standard deviation (SD). Comparisons between two groups were performed with the unpaired Student’s *t*-test, whereas comparison of three or more groups was performed using one-way analysis of variance (ANOVA) with Tukey’s post hoc test. Statistical significance was set at *p* < 0.05 (*p < 0.05, **p < 0.01, ***p < 0.001).

## Supplementary information

Original Data

Supplementary Figure

Supplementary Table

## Data Availability

All data that support the findings of this study are available from the corresponding author upon reasonable request.
