# Supplementary figures and images for: NINJ1-mediated plasma membrane rupture of pyroptotic endothelial cells exacerbates blood-brain barrier destruction caused by neutrophil extracellular traps in traumatic brain injury
Source: Cell Death Discov. 2025 Feb 20;11:69. doi: 10.1038/s41420-025-02350-x (PMC11842820; doi:10.1038/s41420-025-02350-x)

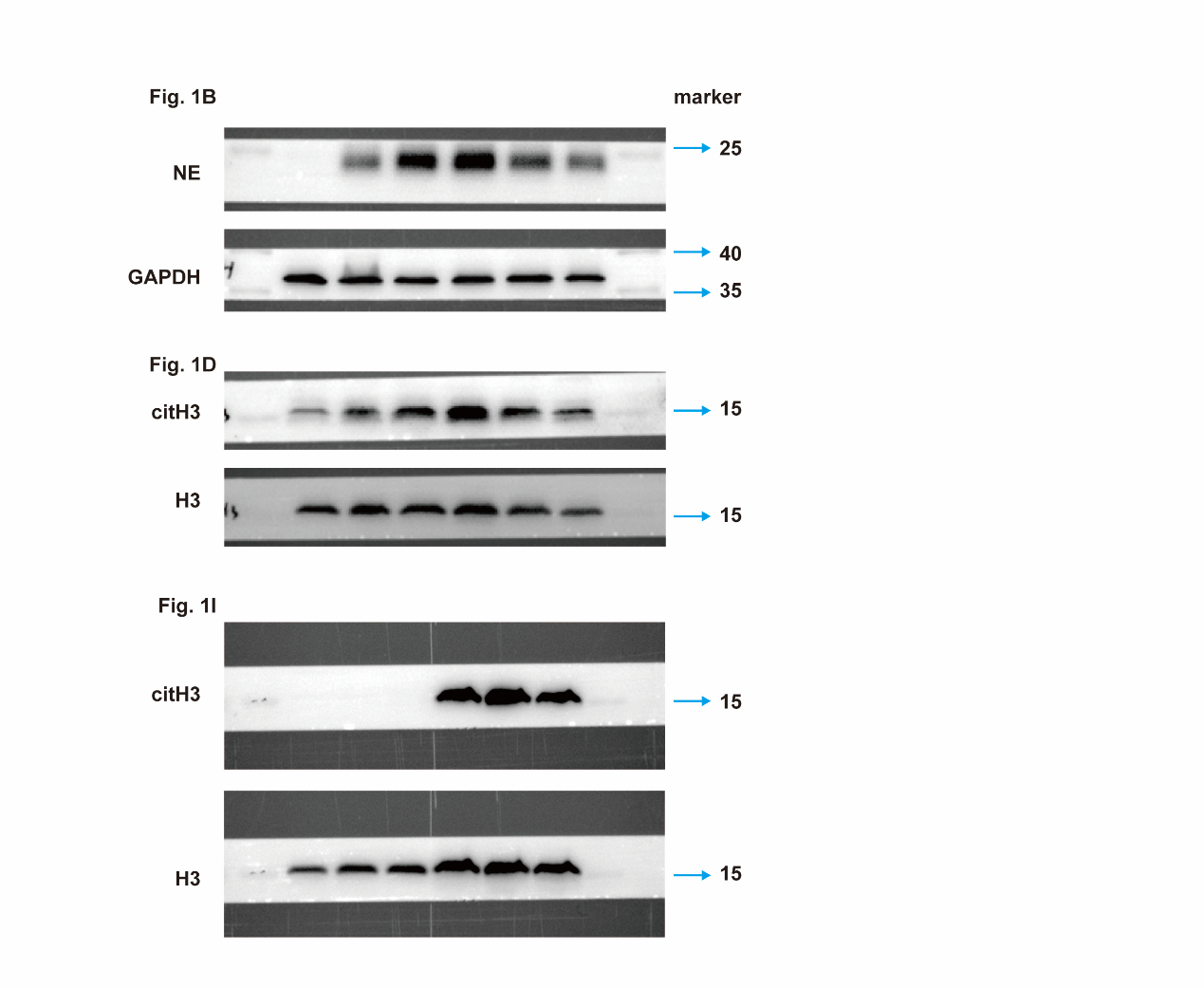


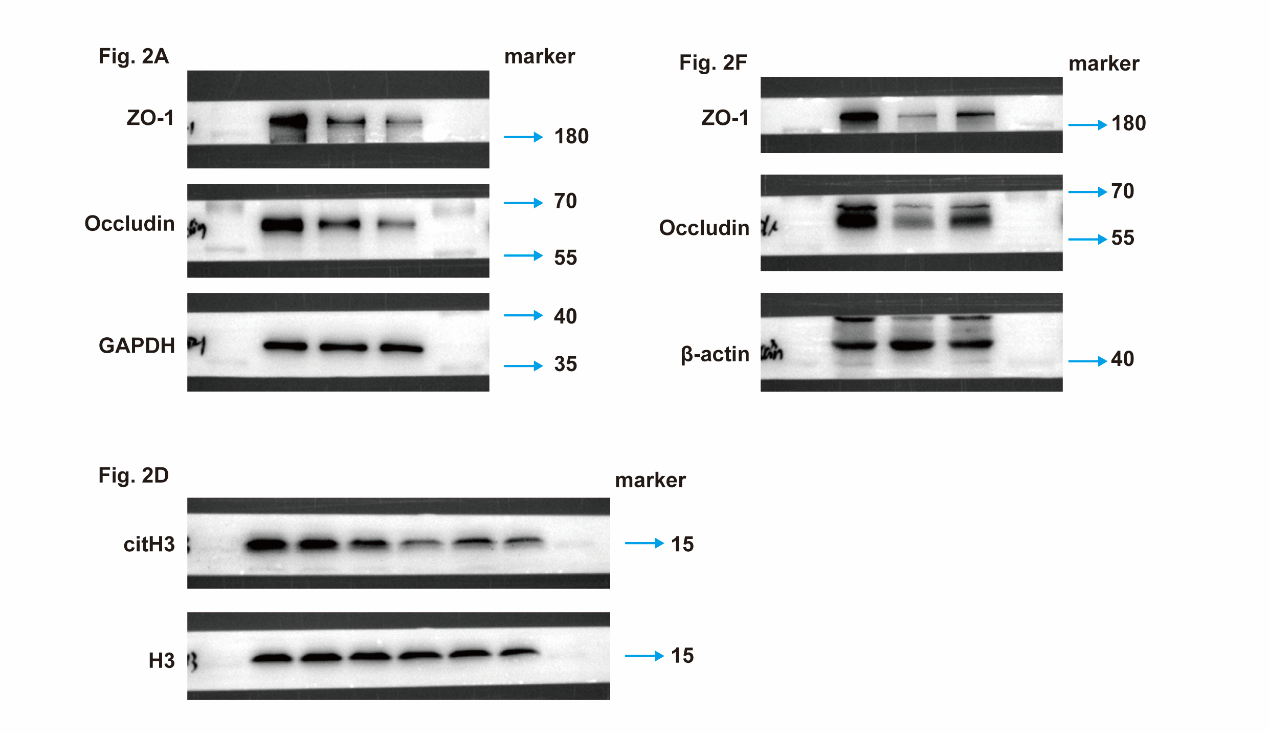


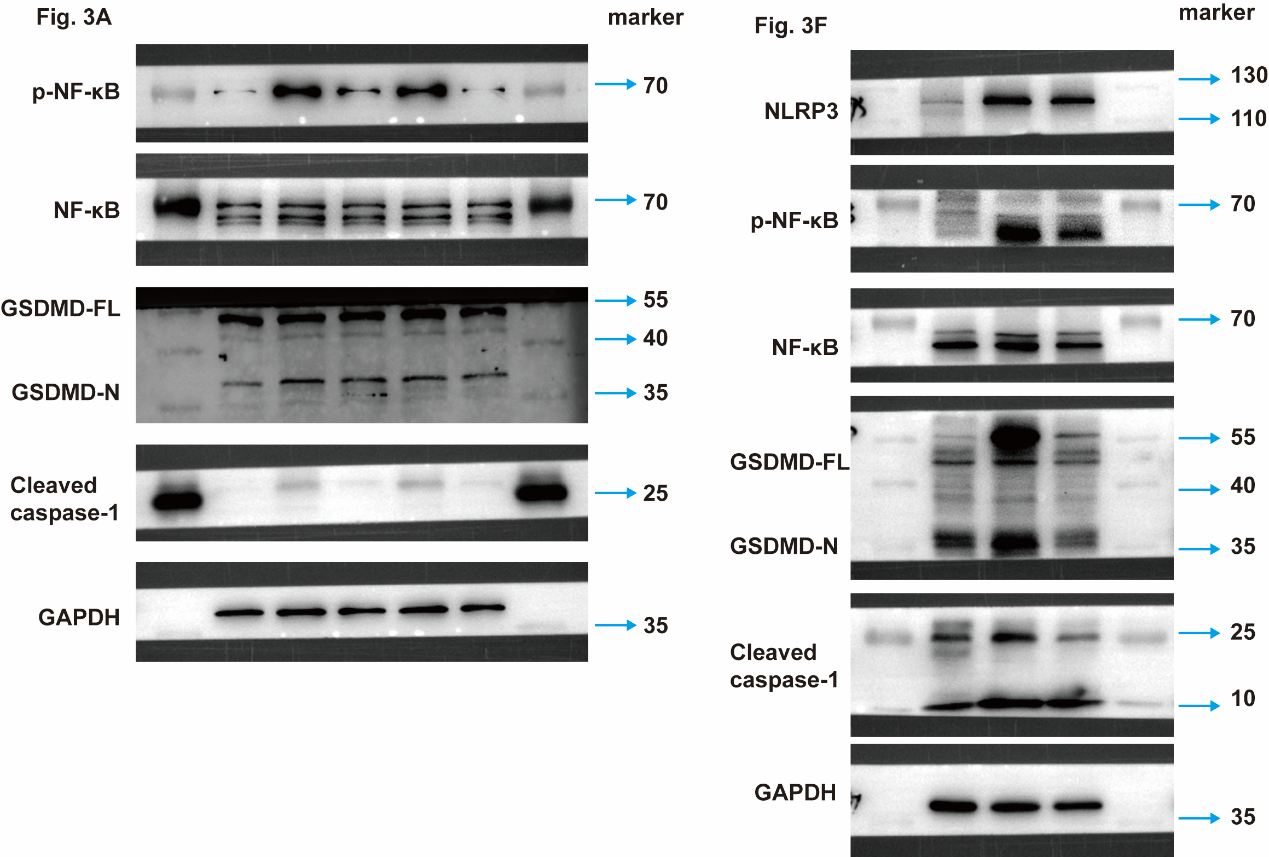


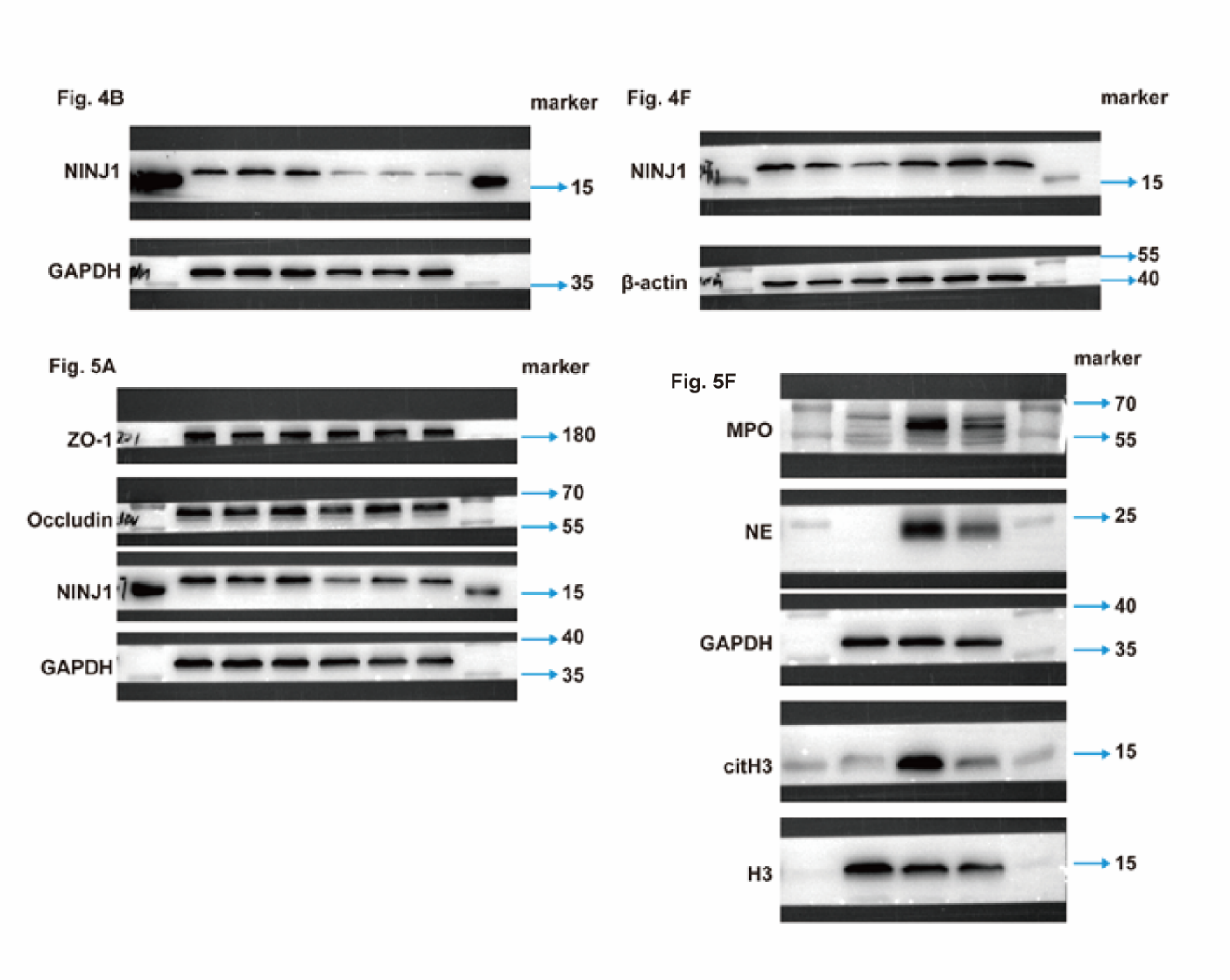


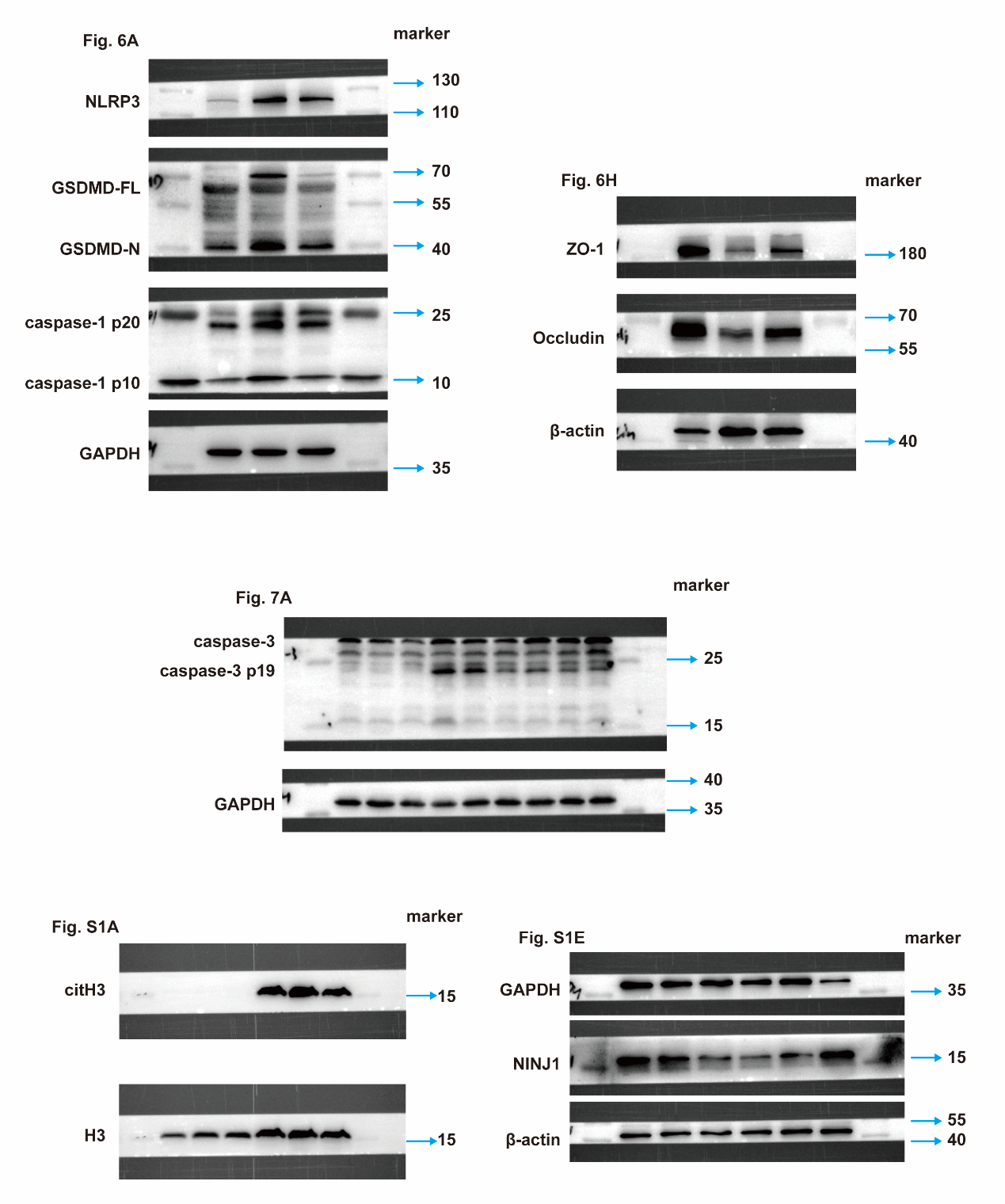


**35**

**55**

**40**

Supplement: Supplementary file 1 — Original Data [file 41420_2025_2350_MOESM1_ESM.docx]
