# Supplementary material for: NINJ1-mediated plasma membrane rupture of pyroptotic endothelial cells exacerbates blood-brain barrier destruction caused by neutrophil extracellular traps in traumatic brain injury
Source: Cell Death Discov. 2025 Feb 20;11:69. doi: 10.1038/s41420-025-02350-x (PMC11842820; doi:10.1038/s41420-025-02350-x)
Supplement: Supplementary file 2 — Supplementary Figure [file 41420_2025_2350_MOESM2_ESM.docx]

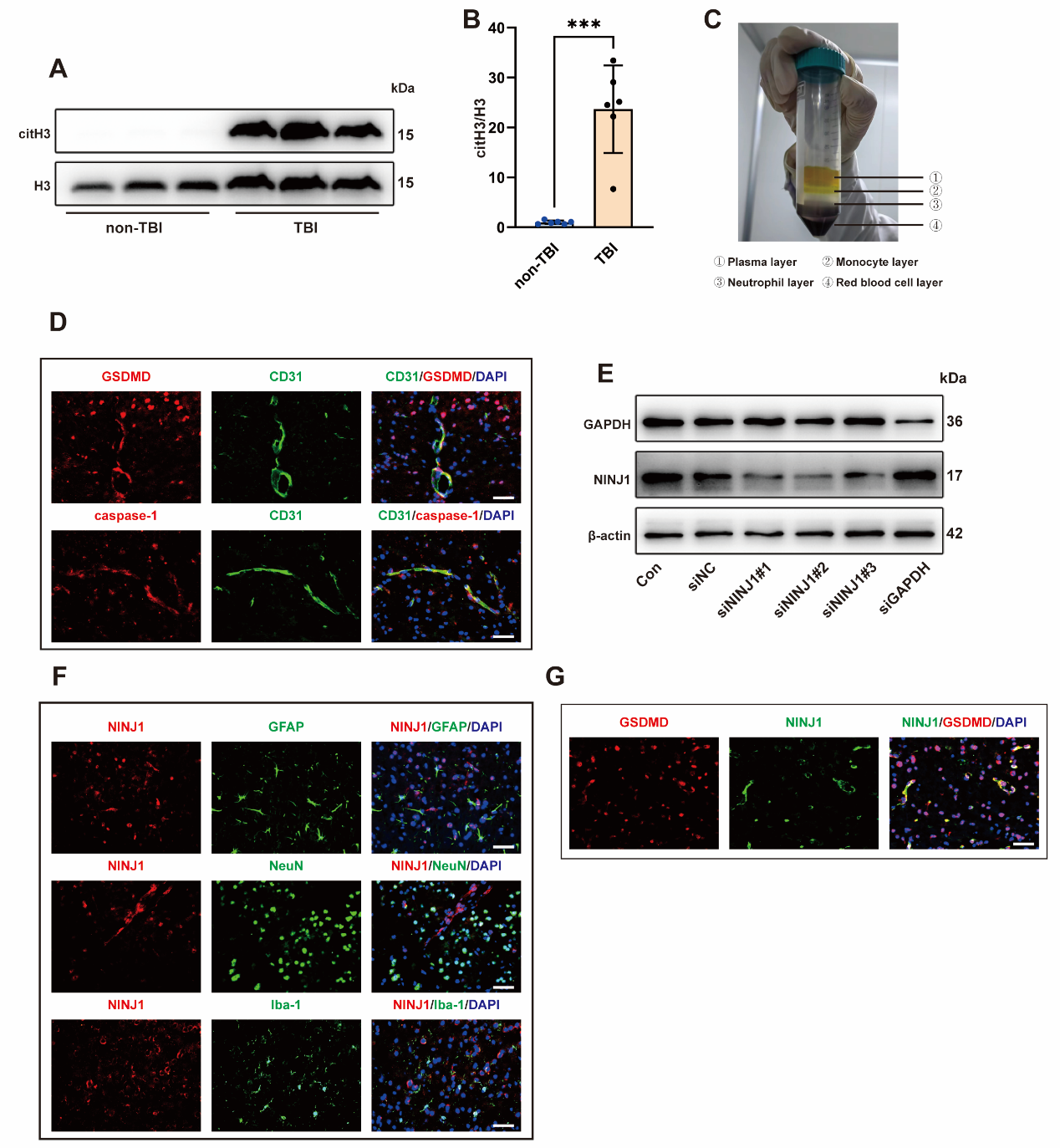


**Supplementary Fig. 1 A–B** Western blotting of the NET-specific marker citH3 in the contused brain tissue of patients with and without TBI (n = 6). **C** Representative image of neutrophil isolation from human blood by gradient centrifugation. **D** Representative image of immunofluorescence staining of CD31 (green) and pyroptosis-related proteins (red, GSDMD and caspase-1) in the cortex. Nuclei were stained with DAPI (blue). Scale bar = 50 μm. **E** Western blotting of NINJ1 in hCMECs treated with three strand siRNAs for NINJ1. **F** Representative image of immunofluorescence staining of NINJ1 (red) with GFAP, NeuN, or Iba-1 (green) in the cortex. Nuclei were stained with DAPI (blue). Scale bar = 50 μm. **G** Representative image of immunofluorescence staining of GSDMD (red) with NINJ1 (green) in the cortex. Nuclei were stained with DAPI (blue). Scale bar = 50 μm. Data are presented as the mean ± SD, ns: Not significant. *p < 0.05, **p < 0.01, ***p < 0.001.
