# Supplementary material for: NINJ1-mediated plasma membrane rupture of pyroptotic endothelial cells exacerbates blood-brain barrier destruction caused by neutrophil extracellular traps in traumatic brain injury
Source: Cell Death Discov. 2025 Feb 20;11:69. doi: 10.1038/s41420-025-02350-x (PMC11842820; doi:10.1038/s41420-025-02350-x)
Supplement: Supplementary file 3 — Supplementary Table [file 41420_2025_2350_MOESM3_ESM.docx]

**Supplementary Table 1. Clinical characteristics of the TBI patients and healthy donors.**

| Characteristics | Healthy donors (n = 6) | Traumatic brain injury  patients (n = 6) | p-value |
| --- | --- | --- | --- |
| Age (mean ± SD; years) | 24.5±1.38 | 48.17±13.29 | 0.0017 |
| Male (%) | 100 | 50 | 0.049 |
| Diabetes (%) | 0 | 0 | 0.999 |
| Asthma (%) | 0 | 0 | 0.999 |
| Hypertension (%) | 0 | 0 | 0.999 |
| Other neurological diseases (%) | 0 | 0 | 0.999 |

**Supplementary Table 2. Clinical characteristics of the TBI and non-TBI patients.**

| Characteristics | Control  (n = 6) | Traumatic brain injury  patients (n = 6) | p-value |
| --- | --- | --- | --- |
| Age (mean ± SD; years) | 58.17±4.22 | 55.83±9.28 | 0.587 |
| Male (%) | 33.33 | 50 | 0.145 |
| Diabetes (%) | 0 | 16.66 | 0.341 |
| Asthma (%) | 16.66 | 0 | 0.341 |
| Hypertension (%) | 33.33 | 16.66 | 0.549 |
| Other neurological diseases (%) | 0 | 0 | 0.999 |
